# Self-identification of Pelvic Floor Disorder Symptoms Among Latina Women: The Roles of Knowledge, Attitude, Behaviors, Beliefs, and Psychosocial Factors

**DOI:** 10.1007/s40615-025-02421-w

**Published:** 2025-06-17

**Authors:** Temitope Rude, Claudia Sevilla, Nanjun Chen, Priscilla Marin, Alexandra Chavez, Nahid Yosufi, Jennifer B. Unger, Lourdes Baezconde-Garbanati, Christina E. Dancz, David Ginsberg, Larissa V. Rodriguez, Mariana C. Stern

**Affiliations:** 1 Department of Urology, Stanford University, Stanford, CA USA; 2 Department of Urology, Keck School of Medicine of USC, University of Southern California, Los Angeles, CA USA; 3 Department of Population and Public Health Sciences, Keck School of Medicine of USC, University of Southern California, Los Angeles, CA USA; 4 Department of Obstetrics and Gynecology, Keck School of Medicine of USC, University of Southern California, Los Angeles, CA USA; 5 Department of Urology, Weill Cornell Medicine, New York, NY USA

**Keywords:** Pelvic floor disorders, Hispanic women, Latinas, Healthcare disparity, Health Knowledge, Attitudes, Practice

## Abstract

**Purpose:**

The connection between experiencing symptoms and identifying them as a pelvic floor disorder (PFD) among Latina women is not well understood. Self-identification of PFD is a critical first step toward timely diagnosis. This study investigated self-reported symptoms of urinary incontinence (UI), fecal incontinence (FI), and pelvic organ prolapse (POP) in community-dwelling Latina women in Los Angeles to assess whether cultural and psychosocial factors influence the self-identification of PFDs.

**Methods and Materials:**

We conducted a cross-sectional survey of community-dwelling Latina women (median age = 50) in Los Angeles using standardized surveys, including measures of acculturation; Latino and mainstream cultural values; knowledge, attitudes, behaviors, and beliefs about PFDs; the Pelvic Floor Distress Index-20 (PFDI-20); and measures of stress and discrimination. Descriptive statistics, univariate, and multivariate logistic regression were performed to identify variables associated with self-identification of PFDs.

**Results:**

Of 197 women, 19% had bothersome symptoms consistent with POP, 33% with UI, and 14% with FI. Among the 70% of women who denied having a PFD, 39% reported at least one bothersome PFD symptom on the PFDI-20. Lower independence, higher religiosity, and higher perceived stress were independently associated with a higher likelihood of PFD self-identification (*p* < 0.05). Higher values of independence were less likely in women who were unsure if they had a PFD (OR = 0.38; 95%CI = 0.17–0.81, *p* = 0.01).

**Conclusions:**

Despite the considerable prevalence of PFD symptoms among Latinas in Los Angeles, most do not self-identify as having a PFD. This discordance is correlated with the severity of symptoms and values of independence and religiosity or stress. Interventions to raise awareness and recognize symptoms are needed to improve self-identification and earlier detection of PFDs among Latinas.

## Introduction

Pelvic floor disorders (PFDs) encompass several conditions that affect nearly 25% of women in the USA [1], most commonly urinary incontinence, pelvic organ prolapse, and fecal incontinence [2]. These disorders cause significant morbidity, both physical and emotional. Furthermore, up to 20% of women in the population are estimated to require operative intervention by age 80 [3].

Latina women have been reported to have higher rates of prolapse and incontinence when compared to other ethnicities in some studies [4–6], although not in others [2, 7]. Moreover, Latina women have been reported to seek care for signs and symptoms related to these disorders much later than women from other racial and ethnic groups [8, 9]. The reasons underlying these disparities are not fully understood. They may partly be due to social stigma, low health literacy about PFDs, or access to care due to financial, logistical, or immigration factors [10–14]. Many Latina women believe PFDs are abnormal and a source of embarrassment [15, 16]; therefore, compared to non-Latina women, they often maintain more secrecy about their condition [8] and prioritize their familial responsibilities above their health needs [14], both of which translate to delays in care. Moreover, language barriers and concerns about provider attitudes have been reported to impair the patient-provider relationship and outcomes for PFD treatment [11, 17, 18]. Health disparities in the Latinx community can be associated with differences in education, immigration, and acculturation, highlighting the importance of accounting for these factors when studying determinants of health disparities within this community [19].

Little is known about how Latina women interpret their PFD-related symptoms and decide to seek care. This process is at the foundation of improving timely access to care for PFD among Latina women, as the most critical step is for women to identify their symptoms as a disorder worthy of seeking care. Symptoms of PFD can be assessed by several validated tools, such as the Pelvic Floor Distress Index (PFDI) which has been validated in a short form of 20 questions [20]. While this questionnaire was initially validated for use in a symptomatic population, it has become more commonly used to assess symptoms outside of the clinical setting to assess overall prevalence of PFD in US women; [21, 22] and higher scores correlate with self-identification of having PFD among women [23].

Guided by the theory of planned behavior [24] as a conceptual framework, which has previously been used to evaluate other health-seeking behaviors [25], our study assessed symptoms of PFDs among Latina women living in Los Angeles and evaluated the medical, cultural, and social factors associated with their identifying those symptoms as a PFD. We evaluated attitudes, subjective norms, and perceived behavioral control that could influence the behavior of identifying PFD symptoms as such, which in turn can prompt seeking care. Among the subjective norms, we included Latino cultural values [26], which can influence the decision of a women to prioritize the needs of their family over the own (“familism”) or the belief that PFD symptoms are part of aging and nothing can be done (“fatalism”) [27]. We hypothesized that higher symptom severity, as measured by a validated scale, would be associated with higher likelihood of self-identification of a PFD. We further hypothesized that self-identification of having a PFD among Latina women with urinary, fecal, or pelvic organ prolapse symptoms could be negatively influenced by increased stress and lower levels of acculturation, which capture integration to US lifestyle.

## Materials and Methods

### Participants

We conducted a cross-sectional study among Latina women in the city of Los Angeles. A community health worker (*promotora de salud*) identified women at health fairs, schools, parks, and community centers throughout the city of Los Angeles between June 2019 and October 2019, and administered surveys, in English or Spanish, on site. All participants were women age 18 and above, self-identified as Latina, and were able to understand the survey in either Spanish or English. Surveys were administered by the *promotora de salud*. The term PFD was defined to participants to include “a bulge in the vagina from the bladder, uterus, or rectum falling out, or involuntary leakage of stool or urine.” Of the 205 women who participated, three had extensive missing data, and five did not respond to the PFD self-identification question, leaving a total of 197 participants in our sample.

### Surveys

Surveys included seven sections: demographics; acculturation; Latino values; knowledge, attitude, behaviors, and beliefs (KABB) about PFDs; PFD symptom survey; sexual function; perceived stress scale; and everyday discrimination. Demographics included patient age, marital status, employment status, income, and level of education.

Acculturation, the degree of assimilation to US culture, was measured via the validated revised Acculturation Rating Scale for Mexican-Americans-II (ARSMA-II) which has been used to study Latinx individuals in the USA [28], based on Berry’s model [29]. The ARSMA-II is a 30 question survey of four acculturation modes: integration, separation, assimilation, and marginalization toward Latin American and US American acculturation. The validated scoring rubric yields both a Latin American Orientation Score (LAOS) and an Anglo Orientation Score (AOS) which range from 0 to 4, with higher scores representing higher acculturation toward Latin American and US American cultures, respectively.

The values section included the Mexican American Cultural Values Scale for Adolescents and Adults [30] and includes 50 questions that assess values of competition, familism, independence, material success, religion, respect, and traditional gender roles, with sub-scores each ranging from 0 to 4. These sub-scores are then grouped into a Latin American Values Score and Mainstream US Values Score, and these summary scores also have a range of 0–4.

The KABB section included 21 questions that we developed for the purpose of this study. The first five questions asked if a woman thought she had a PFD and assessed beliefs regarding the need or importance of seeking care for a PFD (Table 1). The following 16 questions were completed only by women who self-identified as having a PFD and focused on patterns and determinants of seeking care, and effects PFDs had on their self-image and sexuality. All questions were presented in a multiple-choice format and/or a Likert scale, with some having open text options to elaborate as needed.

**Table 1:** Pelvic floor knowledge questions

1. When should a woman get checked for pelvic floor problems?
2. What do you think can help treat pelvic floor problems?
3. Do any of your relatives or friends have pelvic floor problems?
4. In your opinion, how important is it, on a scale from 1 to 10, where 1 means not at all and 10 means extremely important, for women to go to the doctor when they have pelvic floor problems?
5. Do you think you have symptoms of pelvic floor disorders?

Patients were screened for PFD symptoms with the Pelvic Floor Distress Inventory questionnaire (PFDI- 20), which is translated and has been validated for use in Spanish [31]. It is comprised of 20 questions on a Likert scale of 0–4, combined into a Pelvic Organ Prolapse Distress Index (POPDI- 6), Colorectal Distress Index (CRADI- 8), and Urinary Distress Index (UDI- 6) [32]. These indices are scored from 0 to 100, with the total PFDI scored as their sum, from 0 to 300. Sexual function symptoms and beliefs were assessed with the Pelvic Organ Prolapse/Urinary Incontinence Sexual Function Questionnaire (PISQ- 12), which is translated and validated in Spanish [33, 34] and scored from 0 to 48. We assessed overall stress using the Perceived Stress Scale (PSS) [35] which has been translated into Spanish and validated among Latinx, scored 0–40 [36]. We also assess potential stressors due to being Latina and/or immigrants via the Everyday Discrimination Scale (EDS), scored 0–5 [35, 37]. The EDS was reported to perform well among Latinx, except for one construct pertaining to one question (“People act as if they’re better than you are”) which showed lower correlation among Latinx [38].

### PFD Symptom Score Variables

The total PFDI- 20 scores (0–300) and POPDI- 6, CRADI- 8, and UDI- 6 sub-scores (0–100) were used as continuous variables. In addition, we derived the following summary variables: (1) presence of prolapse, bowel, or urinary symptoms, or overall PFD symptoms, defined as at least one response with a score of 2–4 within the POPDI- 6, CRADI- 8, UDI- 6, or PFDI- 20, respectively; (2) presence of bothersome prolapse, bowel, or urinary symptoms, or overall PFD symptoms defined as at least one response with a score of 3–4 within each subgroup or within the PFDI- 20 overall; (3) number of symptoms was defined as the number of questions with at least one response with a score of 2–4; (4) number of bothersome symptoms was defined as number of questions with a score 3–4. For the latter count variables, they were calculated for each subgroup within the PFDI- 20 and for overall PFDI- 20. Presence of PFD, as determined by the participant, was defined as a positive response to the direct question “Do you think you have a PFD” and coded as yes, no, or not sure.

### Statistical Analysis

Our first aim was to evaluate the relationship between PFD symptoms and self-reported PFD. We created three groups of women: those who identified as having a PFD (“identifiers”), those who denied having a PFD (“deniers”), or those who were unsure. We compared frequencies between identifiers, deniers, and unsure women for the following variables: participants’ age, education level (completed primary school, secondary school, college and beyond), employment status (unemployed, homemaker, employed), religion (Catholic, Protestant, other), marital status (married, single, unknown), nativity (US born or foreign born), Latin American Orientation Score (LAOS), Anglo Orientation Score (AOS), Values scores, Everyday Discrimination Score (EDS), and Perceived Psychosocial Stress (PSS) score. We considered as possible determinants the overall PFDI- 20 scores, the POPDI- 6, CRADI- 8, and UDI- 6 as well as the number of bothersome PFDI symptoms. We performed descriptive statistics and univariate analyses via ANOVA or the Kruskal–Wallis test for non-normally distributed variables, for continuous variables and chi-squared testing for categorical variables. Odds ratios (OR) and 95% confidence intervals (CI) from separate logistic regression models were used to identify determinants of positive self-identification versus negative self-identification, and for unsure self-identification versus positive self-identification. Logistic regression models were created in an iterative process, starting with PFDI- 20 scores or number of bothersome PFDI symptoms, as well as any variables that associated with our outcomes with a significance level of *p* ≤ 0.1 on univariate analysis. Only variables that changed estimates of PFDI- 20 scores or number of bothersome PFDI- 20 symptoms by more than 10% or were associated with the outcome at a *p*-value of < 0.1 were kept in the models, as these variables would meet criteria for being potential meaningful confounders.

As secondary sensitivity analyses, we also evaluated the association between level of acculturation and self-identification of PFDs among women who reported symptoms via the PFDI- 20 survey. For these analyses, we only included women with at least one PFD symptom on the PFDI- 20 questionnaire. Analyses were performed as described above, considering self-identification of PFD (yes versus no and yes versus unsure) as outcomes in separate models, and the AOS and MOS scores as key determinants, and considering all other variables as potential determinants and/or confounders as described above.

The variance inflation factor (VIF) was used to assess collinearity among covariates. We considered values higher than 2.5 indicative that a covariate is correlated with the other model covariates. There was no evidence of collinearity in our models. Unless stated otherwise, we considered *p*-values < 0.05 to be significant. All analyses were performed using STATA software, version 15.1 (StataCorp LLC, TX). This study was approved by the University of Southern California Institutional Review Board.

## Results

The median age of participants was 50 years old (range 18–88). Most participants were Catholic (75%), foreign-born (74.5%), had not attended college (73.5%), and stated not knowing their household income (80%). Among foreign-born Latina women (75.5% of women), 58% were from Mexico, 11% from El Salvador, 10% from Guatemala, and the rest were from Peru, Colombia, Brazil, or not reported. The median PFDI score was 30.2 (IQR 7.3–81.3) out of 300, with higher scores indicated increased bother. Other socio-demographic characteristics and scores are summarized Table 2.

**Table 2:** Characteristics of study participants by self-identification of PFD status

Characteristics	All		Deniers		Identifiers		Unsure		Heterogeneity *p*-value		
*N*	197		139	70%	29	15%	29	15%	Overall	Yes vs. no	No vs. unsure
	Median	(Q1, Q3)	Median	(Q1, Q3)	Median	(Q1, Q3)	Median	(Q1, Q3)			
**Demographics**											
Age	50	(37, 61)	49	(36, 60)	57	(46, 68)	48	(39, 56)	**0.039**	**0.015**	0.920
Marital status (*n*, %)									0.360	0.370	0.370
Unknown	8	4.1%	7	5.1%	0	0.0%	1	3.4%			
Married	89	45.6%	61	44.2%	11	39.3%	17	58.6%			
Unmarried	98	50.3%	70	50.7%	17	60.7%	11	37.9%			
Religion (*n*, %)									0.430	0.450	0.350
Catholic	147	75.0%	101	73.2%	24	82.8%	22	75.9%			
Protestant	11	5.6%	9	6.5%	2	6.9%	0	0.0%			
Other	38	19.4%	28	20.3%	3	10.3%	7	24.1%			
Education level (*n*, %)									0.130	0.083	0.170
Primary school	52	26.5%	30	21.7%	11	37.9%	11	37.9%			
Secondary school	92	46.9%	66	47.8%	14	48.3%	12	41.4%			
College	52	26.5%	42	30.4%	4	13.8%	6	20.7%			
Nativity (*n*, %)									0.380	0.220	0.400
Foreign born	146	74.5%	99	71.7%	24	82.8%	23	79.3%			
All others	50	25.5%	39	28.3%	5	17.2%	6	20.7%			
Employment (*n*, %)									0.150	0.170	0.330
Unemployed	69	35.0%	48	34.5%	15	51.7%	6	20.7%			
Homemaker	43	21.8%	29	20.9%	6	20.7%	8	27.6%			
Employed	85	43.1%	62	44.6%	8	27.6%	15	51.7%			
**Symptom assessment**											
*Survey scores*^a^											
Pelvic Floor Dysfunction Inventory (PFDI- 20)	30	(7, 81)	21	(4, 59)	71	(13, 135)	78	(42, 94)	** < 0.001**	**0.002**	** < 0.001**
Pelvic Organ Prolapse Distress Inventory (POPDI- 6)	4	(0, 25)	0	(0, 21)	21	(0, 42)	25	(13, 33)	** < 0.001**	**0.006**	** < 0.001**
Colorectal-Anal Distress Inventory (CRADI- 8)	6	(0, 25)	3	(0, 22)	9	(0, 31)	16	(0, 25)	**0.014**	**0.018**	**0.034**
Urinary Distress Inventory (UDI- 6)	13	(0, 38)	13	(0, 25)	46	(8, 50)	29	(8, 50)	** < 0.001**	** < 0.001**	** < 0.001**
*Survey summary (count per participant)*^*b*^											
Number of symptoms	2	(0, 5)	1	(0, 3)	7	(0, 11)	5	(0, 8)	** < 0.001**	** < 0.001**	**0.003**
Number of prolapse symptoms	0	(0, 1)	0	(0, 1)	0	(0, 3)	1	(0, 2)	** < 0.001**	**0.004**	** < 0.001**
Number of bowel symptoms	0	(0, 1)	0	(0, 1)	0	(0, 1)	1	(0, 2)	**0.013**	**0.015**	**0.032**
Number of urinary symptoms	1	(0, 3)	0	(0, 1)	0	(0, 1)	2	(0, 4)	** < 0.001**	** < 0.001**	**0.003**
Number of bothersome symptoms	0	(0, 2)	0	(0, 1)	1	(0, 4)	0	(0, 3)	**0.004**	**0.003**	**0.040**
Number of bothersome prolapse symptoms	0	(0, 0)	0	(0, 0)	0	(0, 1)	0	(0, 1)	** < 0.001**	** < 0.001**	**0.002**
Number of bothersome bowel symptoms	0	(0, 0)	0	(0, 0)	0	(0, 0)	0	(0, 0)	0.086	**0.040**	0.180
Number of bothersome urinary symptoms	0	(0, 1)	0	(0, 1)	1	(0, 2)	0	(0, 2)	**0.018**	**0.005**	0.490
*Count of women with reported symptoms*^*b*^* (n, %)*											
≥ 1 symptom on PFDI- 20	125	63.5%	83	59.7%	21	72.4%	21	72.4%	0.24	0.20	0.20
≥ 1 symptom on POPDI- 6	71	36.0%	40	28.8%	14	48.3%	17	58.6%	**0.003**	**0.041**	**0.002**
≥ 1 symptom on CRADI- 8	75	38.1%	45	32.4%	15	51.7%	15	51.7%	**0.039**	**0.048**	**0.048**
≥ 1 symptom on UDI- 6	104	52.8%	65	46.8%	20	69.0%	19	65.5%	**0.031**	**0.030**	0.066
≥ 1 bothersome symptom on PFDI- 20	76	38.6%	45	32.4%	17	58.6%	14	48.3%	**0.016**	**0.008**	0.10
≥ 1 bothersome symptom on POPDI- 6	37	18.8%	16	11.5%	11	37.9%	10	34.5%	** < 0.001**	** < 0.001**	**0.002**
≥ 1 bothersome symptom on CRAD- 8	28	14.2%	15	10.8%	7	24.1%	6	20.7%	0.097	0.053	0.14
≥ 1 bothersome symptom on UDI- 6	65	33.0%	41	29.5%	15	51.7%	9	31.0%	0.066	**0.021**	0.87
**Acculturation**											
Anglo Orientation Score (AOS)	1	(1, 3)	2	(1, 3)	1	(0, 2)	2	(1, 2)	0.310	0.180	0.740
Latin American Orientation Score (LAOS)	4	(3, 4)	4	(3, 4)	3	(2, 4)	3	(3, 4)	**0.003**	**0.004**	**0.023**
**Values**											
Familism	3	(3, 4)	3	(3, 4)	3	(3, 4)	3	(3, 3)	0.380	0.370	0.210
Respect	3	(3, 4)	3	(3, 4)	3	(3, 4)	3	(3, 3)	0.270	0.360	0.140
Religion	4	(3, 4)	4	(3, 4)	4	(3, 4)	4	(3, 4)	0.350	0.950	0.170
Gender roles	2	(1, 3)	2	(1, 3)	2	(1, 3)	2	(1, 3)	0.540	0.350	0.460
Material success	1	(1, 2)	1	(1, 2)	2	(1, 2)	1	(1, 2)	0.220	0.200	0.340
Independence	3	(3, 3)	3	(3, 4)	3	(3, 3)	3	(2, 3)	**0.017**	0.200	**0.007**
Competition	3	(2, 4)	3	(2, 4)	3	(2, 3)	3	(2, 3)	0.480	0.960	0.240
Latin American values	3	(2, 3)	3	(2, 3)	3	(2, 3)	3	(3, 3)	0.730	0.490	0.870
Mainstream US values	2	(2, 3)	2	(2, 3)	3	(2, 3)	2	(2, 2)	0.096	0.700	**0.045**
**Psychosocial assessment**											
Everyday discrimination	1	(0, 2)	1	(0, 2)	0	(0, 2)	1	(0, 2)	0.760	0.470	0.930
Perceived stress	15	(12, 19)	14	(11, 18)	19	(15, 20)	16	(15, 21)	**0.006**	**0.017**	**0.013**

^a^The Pelvic Floor Distress Inventory (PFDI- 20) questionnaire is comprised of 20 questions on a Likert scale of 0–4, combined into a Pelvic Organ Prolapse Distress Index (POPDI- 6), Colorectal Distress Index (CRADI- 8), and Urinary Distress Index (UDI- 6) [32]. These indices are scored from 0 to 100, with the total PFDI scored as their sum, from 0 to 300. ^b^Each question answered with a Likert score of 2–4, representing at least mild bother, is defined as a present symptom. Each question with a Likert score of 3–4, which represents moderate-severe bother, is defined as a bothersome symptom

**Fig. 1:**
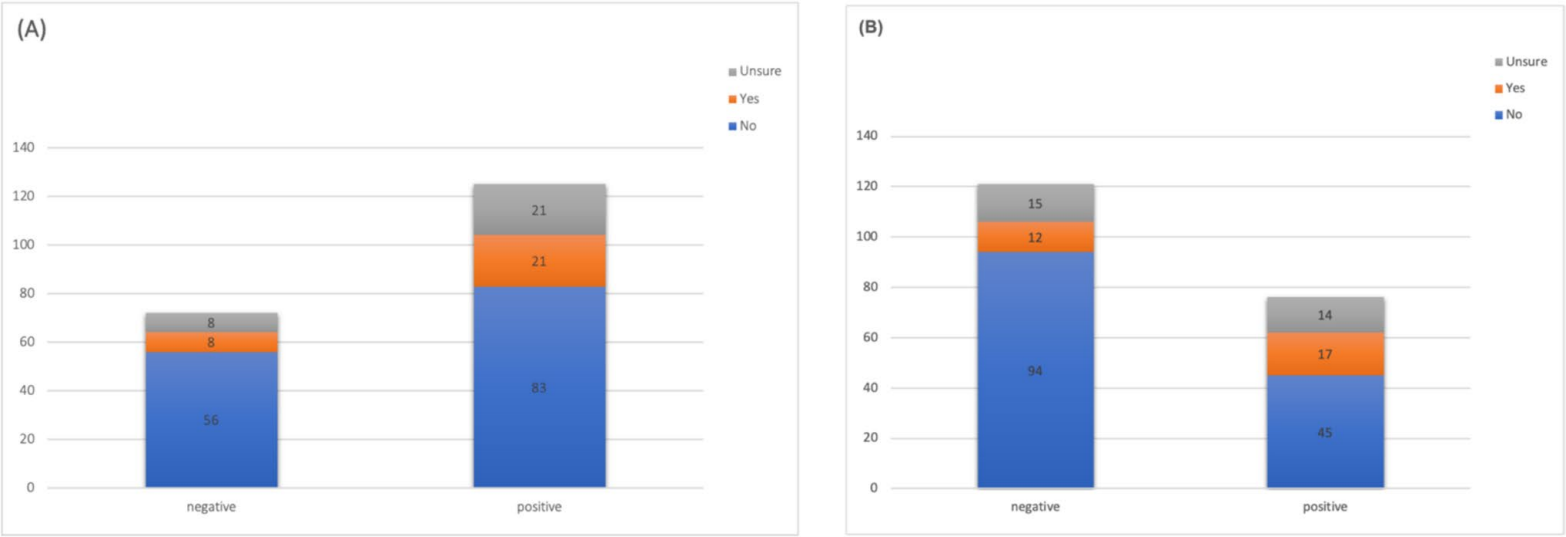
Frequency of self-identification of PFD symptoms stratified by response to PFDI questionnaire. **A** Presence of any symptoms on PFDI. **B** Presence of bothersome symptoms on PFDI

Few women with symptoms based on the PFDI- 20 correctly identified those symptoms as suggestive of a PFD. Only 29 women (15%) answered yes to the question “Do you think you have symptoms of PFDs,” whereas 139 women (70%) answered no, and 29 women (15%) were unsure. We refer to the 29 women who identified as having a PFD as “identifiers,” the 139 women who denied having a PFD as “deniers,” and the 29 women who were unsure as “unsure.” However, 125 responders (63%) reported at least one PFD symptom on the PFDI- 20, with 76 (39%) reporting at least one bothersome symptom, with moderate or severe bother reported. Among the 139 women who did not think they had symptoms of PFDs, 60% reported at least one PFD symptom elsewhere in the PFDI, and 32% had at least one bothersome symptom. The relationship between self-identification of having a PFD and symptoms of PFD as assessed on the PFDI are shown in Fig. 1.

We analyzed differences and associations among these three groups of women based on their self-identification of PFD, without respect to symptoms endorsed on the PFDI. There were significant differences across several domains between these groups (Table 2). Deniers or those who were unsure were significantly younger than identifiers (*p* < 0.05). When considering psychosocial variables, differences were observed for the Latin American orientation score, value of Independence, Mainstream US values, and the Perceived Stress Scale (Table 2). There were no significant differences between groups in marital status, religious affiliation, educational attainment, nativity, or employment status (Table 2).

When considering symptom assessment, identifiers had significantly higher overall PFDI scores than deniers (71 vs. 21, *p* < 0.002), and those who were unsure the highest median score of all at 78, statistically similar to the scores of the identifiers while significantly higher than the scores of the deniers (*p* < 0.001, Table 2). POP, CRAD, and UDI subscores, reflecting pelvic prolapse, colorectal, and urinary incontinence symptoms were similar between identifiers and women who were unsure (21 vs. 25, 9 vs. 16, 46 vs. 29); both of these groups had significantly higher subgroup scores than deniers (0, 3, 13; *p* < 0.05) (Table 2). In univariate analyses, the reported presence of POP was significantly associated with identifiers (OR = 2.3; 95% CI = 1.02–5.22) or those who were unsure (OR = 3.5; 95% CI = 1.54–8.00). Similarly, the presence of UI symptoms was also significantly associated with identifiers (OR = 2.5; 95% CI = 1.08–5.94), with similar association for those who were unsure, although it was not statistically significant (Table 3). The presence of bothersome PFD symptoms was statistically significantly associated with identifiers (OR = 3.0; 95% CI = 1.30–6.72) (Table 3). For symptom-specific scores, both bothersome POP (OR 4.7, 95% CI 1.9–11.7) and bothersome UI symptoms (OR = 2.6; 95% CI = 1.13–5.78) were positively associated with indentifiers, with POP symptoms being also associated with those who were unsure (Table 3).

**Table 3:** Univariate estimates of association between PFDI scores and self-identification of PFD (*p < 0.05)

	Identifiers vs. deniers						Unsure vs. deniers					
	OR	CI		*p*-value	Sensitivity	Specificity	OR	CI		*p*-value	Sensitivity	Specificity
PFDI result		Low	High					Low	High			
> 1 Symptom on the PFDI	1.8	0.73	4.28	0.192	0.72	0.40	1.8	0.73	4.28	0.192	0.72	0.4
> 1 Symptom on the POPDI	2.3	1.02	5.22	0.046	0.48	0.71	3.5	1.54	8.00	0.003	0.59	0.71
> 1 Symptom on the CRAD	2.2	1.00	5.03	0.052	0.52	0.68	2.2	1.00	5.03	0.052	0.52	0.68
> 1 Symptom on the UDI	2.5	1.08	5.94	0.030	0.69	0.53	2.2	0.94	4.99	0.064	0.66	0.53
> 1 Bothersome symptom on the PFDI	3.0	1.30	6.72	0.009	0.59	0.68	2.0	0.87	4.38	0.110	0.48	0.68
> 1 Bothersome symptom on the POPDI	4.7	1.88	11.71	0.001	0.38	0.88	4.1	1.60	10.21	0.004	0.34	0.88
> 1 Bothersome symptom on the CRAD	2.6	0.96	7.19	0.070	0.24	0.89	2.2	0.76	6.14	0.167	0.21	0.89
> 1 Bothersome symptom on the UDI	2.6	1.13	5.78	0.024	0.52	0.71	1.1	0.45	2.56	0.870	0.31	0.71

On multivariate analysis, the key determinants of self-identification of PFD were the number of bothersome symptoms, age, and acculturation as captured by the Latin American Orientation score (LAOS) (Table 4). Specifically, for each additional bothersome PFDI symptom, the odds of being an identifier increased by approximately 20% compared to women who denied having PFD. Increased age and higher LAOS were positively and inversely associated, respectively (Table 4). When comparing women who were unsure to deniers, there was a positive association with the number of bothersome PFDI which did not reach statistical significance, as well as positive associations with the value of religion and perceived stress, and a negative association with the value of independence, which were statistically significant (Table 4).

**Table 4:** Multivariate estimates of association between PFDI scores and self-identification of PFD among all women (*n* = 197)

	Identifiers vs. deniers				Unsure vs. deniers			
	OR	***p***-value	Lower limit	Upper limit	OR	***p***-value	Lower limit	Upper limit
Number of bothersome symptoms	1.2	0.004	1.055	1.339	1.1	0.158	0.954	1.333
Age	1.0	0.018	1.006	1.063	–-	––	––	––
Latin American Orientation score	0.5	0.014	0.312	0.875	–-	––	––	––
Value of religion	–-	––	––	––	2.8	0.015	1.220	6.392
Value of independence	–-	––	––	––	0.3	0.003	0.171	0.706
Perceived Stress Scale	–-	––	––	––	1.1	0.042	1.004	1.198

We completed a subgroup analysis limited to women who endorsed at least one PFD symptom that was at least sometimes bothersome. This subgroup allowed us to more closely study the relationship of associated factors in self-determination of PFD, beyond the presence of symptoms (Table 5). We observed similar associations with self-identification of PFDs as for all women combined reported in Table 4. Specifically, when comparing Identifiers to those who were unsure, we observed that odds of self-identification increased with the number of bothersome symptoms and age, and decreased with increasing LAOS scores. When comparing women who were unsure to deniers, we observed that odds of self-identification increased with higher scores in the value of religion and decreased with higher scores in value of independence. There was a marginally statistically association with increased AOS scores, suggesting that chances of self-identification increased with higher values.

**Table 5:** Multivariate estimates of association between PFDI scores and self-identification of PFD among women with symptoms (*n* = 125)

	Identifiers vs. deniers				Unsure vs. deniers			
	OR	*p*-value	Lower limit	Upper limit	OR	*p*-value	Lower limit	Upper limit
Number of bothersome symptoms	1.2	0.009	1.043	1.352	1.0	0.888	0.817	1.191
Age	1.0	0.036	1.003	1.083	–-	–-	–-	–-
Latin American Orientation score	0.5	0.046	0.279	0.989	1.1	0.786	0.436	3.001
Anglo Orientation score	1.1	0.800	0.610	1.900	1.9	0.059	0.977	3.584
Value of religion	–-	–-	–-	–-	4.0	0.015	1.309	12.343
Value of independence	–-	–-	–-	–-	0.2	0.003	0.067	0.574

## Discussion

In this study, the prevalence of symptoms compatible with PFDs among community dwelling Latina women, as determined by the PFDI- 20, was significantly higher than the prevalence of women who self-identified as having a PFD. Whereas 15% of women recognized having PFDs, and 15% were unsure, we observed that 63% reported at least one PFD symptom and 39% reported at least one bothersome symptom (moderate to severe symptom). Specifically, 19% had symptoms compatible with bothersome POP, 33% with bothersome UI, and 14% with bothersome FI symptoms. Among the 70% of women in this study who denied having a PFD when asked if they thought they had one, 60% reported having at least one PFD symptom on the PFDI- 20, and 32% endorsed at least one bothersome symptom. We observed that the amount of bothersome PFDI- 20 symptoms, age, and the degree of identification with Hispanic culture were statistically significantly associated with self-identification of PFD. Moreover, we found that higher value of religion and lower value independence, as well as high perceived stress scores were statistically significantly associated with being unsure about having a PFD. Our findings are consistent with previous reports that show that Black and other minority women have less PFD knowledge than white women [39].

The prevalence we observed for bothersome PFD symptoms of 39% is comparatively higher than what has been previously reported in a large cross-sectional study of adult women participating in the National Health and Nutrition Examination Survey (NHANES), given our median age of 50 [2]. Similarly to our study, the NHANES study included women ages 20–80 + years old, and reported prevalence for having at least one symptom of 24% for all women, 10% for 20–39 years old, 27% for 40–59 years old, 37% for 60–79 years old, and 50% for women older than 80 years old. When we looked at the same question this NHANES study considered for POP, 31% of women in our study reported a positive response to this same question, compared with only 3% of women in the NHANES study overall. In our study, 7% of participants endorsed bothersome prolapse by this measure. Of the 20% of NHANES participants who identified as Latina, 16% of women endorsed UI, 5% endorsed FI, and 5% endorsed POP. In contrast, we observed that 36% of women had at least one symptom of POP, 53% had at least one symptom of UI, and 38% had at least one symptom of FI symptoms. Our observed prevalence is comparable to what was reported from two large studies of community dwelling women in California that reported a PFD prevalence of 34–37%; however, these studies included a greater proportion of older women than our study and did not use the same instrument to identify PFDs. Therefore, comparisons should be considered with caution [4, 40].

Our observed prevalence for POP is within the higher range of what has been reported by other studies that included Latina women, which range between 5 and 47%, with lower prevalence for studies that used self-report, and higher prevalence for studies with surveys and/or examination [1, 4, 40–42]. The median age across these studies is greater than ours by 5–15 years. Prevalence of UI among Latina women has been reported to range between 7 and 56%, depending of age distribution and how questions about incontinence were asked [43–45], and prevalence of FI has been reported by be 5% [2].

We observed that PFDI- 20 symptom scores were statistically significantly associated with the likelihood of self-identifying PFDs, particularly bothersome POP and UI symptoms. When considering other associations, each additional bothersome PFD symptom increased the likelihood of self-identifying a PFD by 20%. When controlling for age, another determinant of self-identification was the degree of acculturation, suggesting that the experience and recognition of symptoms is influenced by the patient’s cultural context. Among women who were unsure of having a PFD, key determinants of being unsure were the values of independence and religion, and perceived stress. We observed that having a low sense of self-sufficiency and self-reliance, considering that God and prayer is an important presence in their lives, and having higher perceived stress levels, increased the risk that a woman would be unsure about having a PFD. To our knowledge, there have been no other studies that examined the role of these specific Latino values using the same scale we used here in relation to the perception of having PFD, so we cannot compare our findings directly. However, there have been focus groups of Latina women discussing their attitudes toward PFDs, and key themes that emerged are lack of information, shame, and feelings of isolation, with Latinas maintaining more secrecy about having a PFD, being more likely to think that PFDs are a common part of aging, and delaying seeking care compared to other racial/ethnic groups [8, 15, 18]. We can speculate that women with low self-reliance and self-sufficiency might be more likely to accept the symptoms as part of aging, and be less likely to label them as “disease,” seek information, and ultimately seek care.

Social stress has been clearly identified as an important determinant of health and healthcare outcomes among other minorities, including in the Black community [37]. In our study, we observed that higher perceived stress scores (PSS) increased the chance that a woman would be unsure about having a PFD. Further studies are needed to understand what other characteristics not considered here may be correlated with PSS that may explain this finding.

A small subset of women (4%) reported they thought they had a PFD without reporting any symptoms on the PFDI- 20 questionnaire. We speculate this is because the PFDI- 20 does not cover all domains of PFDs. Most notably, these women could be experiencing isolated sexual dysfunction, pelvic pain, gynecologic, or urinary infections. We cannot discard the possibility that perhaps these few women did not understand the provided definition of a PFD. Most of the women with PFD symptoms and negative PFDI- 20 answers did report sexual dysfunction. More studies are needed to follow-up on this and fully explore these factors. The fact that this is a small proportion of the sample is reassuring that the domains of prolapse, bowel symptoms, and urinary incontinence are the dominating categories for PFD in this study.

The strengths of this study include its focus on Latina women enrolled directly from the community, rather than women presenting to a specialty clinic, using a cross-sectional design to capture various communities across the city. This study is the first to specifically investigate factors associated with the likelihood of self-identifying PFDs in a large Latina population, including measures across various domains such as demographics, Latino cultural values, acculturation, stress, and clinical indicators of PFDs, using instruments validated for use among Latino populations. This work is also strengthened by the availability of surveys in both Spanish and English, and the use of a *promotora de salud* to conduct the surveys. Among the weaknesses of our study is the use of the PFDI- 20 in a population without known PFDs, although the PFDI- 20 has been used in the community setting successfully, and the lack of a physical examination to confirm the presence or absence of POP. Follow-up studies that include this in addition to our measurements are needed to validate our findings. Another limitation is the lack of reliable income data among our participants and additional metrics of socioeconomic status (SES). Incorporation of these data would improve the interpretation of our findings, as it has been previously shown that some degree of PFD knowledge is related to SES more than race [46]. As an exploratory and community-based survey study, our study was designed to assess the knowledge and attitudes of a wide range of participants. Whereas income data were collected in the questionnaire, the validity was poor with many women stating that they did not know their income. We felt that requiring accurate and complete income assessments and additional measures of SES would limit this primary aim. For this reason, we did not include socio-economic status data in this analysis. However, given the places where women were recruited, we conclude that the variance of socio-economic status was narrow, and generally SES was low. Further study designs would benefit from incorporation of SES assessments to build upon these data. Another possible limitation of this study is the representativeness of the sample for Latina women in Los Angeles. It is possible that women who agreed to participate may have greater pelvic health concerns than the broader Latina population. We also excluded women who were unable to complete the survey which may impact the representativeness of the sample. We accounted for this by utilizing numerous sites and recruiting nearly 200 women. Importantly, this study describes the population in LA area where Mexican American and Central American women comprise most of the Latino population, and it is not a representative sample of all Latina in the USA. Given the heterogeneity of the Latino populations, culture, racial, and geographic diversity, it remains to be evaluated if these findings will be generalizable to other Latina populations across the USA.

In summary, our findings suggest that the psychosocial context is very relevant in determining whether a Latina woman recognizes she has a PFD and thus may significantly influence whether and when a Latina woman seeks care for these symptoms. Importantly, the factors that significantly differed between women who were unsure if they had a PFD and those who denied having a PFD were not only based on symptom severity, but also based on cultural values, specifically increased values of religiosity and decreased values of independence, and psychosocial factors of perceived stress. Community outreach interventions should be particularly attuned to differences in values to teach Latina women about PFD symptoms and treatment options most effectively. Respecting and understanding the intersection of cultural values and psychosocial factors, such as perceived stress, will better enable interventions to empower women to listen to their bodies and discuss their symptoms with their doctors during their annual check-ups. This may require partnering with community religious or social organizations to improve insight into the needs of the community. Moreover, interventions to raise awareness among healthcare providers about cultural values and social factors that may influence Latina women to disclose and seek care for PFD symptoms would also be appropriate. Together, these interventions could lead to a decrease in disparities in receipt of care. These findings underscore the importance of considering an individual’s social and cultural context in trying to understand their disease burden and their awareness of disease, which will impact their ability to seek care and the impact of disease on their health-related quality of life.

## Data Availability

Available at the University of Southern California.
